# Group size and aquatic vegetation modulates male preferences for female shoals in wild zebrafish, *Danio rerio*

**DOI:** 10.1038/s41598-020-80913-x

**Published:** 2021-01-13

**Authors:** Aditya Ghoshal, Anuradha Bhat

**Affiliations:** Department of Biological Sciences, Indian Institute of Science Education and Research (IISER) Kolkata, Mohanpur, West Bengal 741 246 India

**Keywords:** Ecology, Behavioural ecology

## Abstract

Shoaling decisions in the wild are determined by a combination of innate preferences of the individual along with the interplay of multiple ecological factors. In their natural habitat as well as in the laboratory, zebrafish is a shoaling fish. Here, we investigate the role of group size and associated vegetation in shaping shoaling preferences of wild male zebrafish. We studied the association preference of males to groups of female shoals in a multi-choice test design. We found that males made greater proportion of visits to an 8-female group compared to 2 and 4-female groups. However, males spent similar proportions of time across the three female-containing groups. When artificial vegetation was incorporated along with female number as an additional factor, we found that males prefer high and moderately vegetated patches compared to low or no-vegetation groups, irrespective of the number of females in these patches. Based on experiments using a novel multi-choice design, our results show that preference for group size can change due to interaction of two separate factors. This work is a first attempt to understand the role of aquatic flora in determining shoaling preferences in zebrafish, using an experimental paradigm consisting of a gradation in female and vegetation densities.

## Introduction

Shoaling has been defined as an aggregatory behavior giving rise to a non-random distribution of conspecifics in a given space^1^. In their natural habitat as well as in the laboratory, zebrafish is a shoaling fish living in group sizes ranging from a few individuals to a few hundred^2^. Shoaling allows for efficient foraging and reduced predation risk while also providing easier access to mates^3^. Shoaling provides several survival benefits to the individuals comprising a shoal. Primarily, shoaling reduces predation risk by dilution effect and can also allow for early detection of an approaching predator. Shoaling fish also have better foraging opportunities due to higher chances of food detection but may also increase competition among the individuals for the food resource. This can be balanced by modulating the inter-individual distances between the members, making the shoal less tight^4^.

Shoaling behavior of zebrafish have been traced ontogenically to arise during larval development^3,5^. It has been shown that zebrafish shoals can use visual cues to transmit social information of an immediate predator to group members^6^. Zebrafish individuals prefer to shoal with individuals of the similar phenotype, possibly preventing oddity effect for avoiding predation^7^.

In fish species like guppies (*Poecilia reticulata*), males prefer female-dominated or all-female groups over mixed groups as their chances of finding a suitable mate are higher^8^. Studies on zebrafish indicate that sex influences shoaling preferences with clear differences existing between the two sexes. Zebrafish females are known to choose larger shoals^9,10^ but no such preference for group size is shown to exist among the males. Based on group size, male zebrafish do not distinguish between all male groups^9^. There are also evidences for sex-assortative shoaling in zebrafish females^11^. Females prefer to shoal with other females in choice experiments as well as in free-swimming condition. On the other hand, male zebrafish prefer groups of females over males^9^, and even locations previously inhabited by them (in absence of the visual cues^12^). But males do not show preference for greater female number^9^. Thus, sex and group size seem to strongly influence shoaling preferences. In our current study, we use a novel multi-choice experimental setup to explore how male association with female shoals is shaped by the group size as well as presence of vegetation cover in wild-caught populations.

Aquatic vegetation is an important ecological feature that is known to regulate crucial life history traits like foraging behavior of predators (for example, in Spotted gar *Lepisosteus oculatus*)^13^ and also provide protection and shelter to prey species^14^. Vegetation patches have been shown to modulate the area of foraging territory defended by convict cichlids against intruder fishes^15^. Underwater vegetation is a crucial ecological feature of zebrafish natural habitats. In nature, zebrafish are found in shallow waters, closer to the banks with dense aquatic vegetation^2^. After hatching, zebrafish larvae lack a swim bladder. The hatchlings attach themselves to underside of leaves till swim bladder develops^16^. In the laboratory, zebrafish are known to prefer enriched vegetated areas compared to bare tanks^17,18^. It is also known to influence behavioral traits like aggression in this species^19,20^. Our work attempts to understand the interplay of shoal size and vegetation influencing shoaling decisions in males.

Specifically, our study addressed the following questions.

- Does varying number of receptive females influence the preference/choice of a patch by the males and thus influencing the association pattern?
- Does vegetation density associated with female patches (varying in number) change the association pattern?

We expected the males’ association to a patch would be correlated to the number of females present in that patch. We also expected that in presence of vegetation they would prefer patches with higher degree of vegetation density associated with higher number of females.

## Materials and methods

### Ethics statement

The study complied with the existing rules and guidelines outlined by the Committee for the Purpose of Control and Supervision of Experiments on Animals (CPCSEA), Government of India, the Institutional Animal Ethics Committee’s (IAEC) and guidelines of Indian Institute of Science Education and Research (IISER) Kolkata. All experimental protocols followed here have been approved by the Institutional Animal Ethics Committee’s (IAEC) and guidelines of Indian Institute of Science Education and Research (IISER) Kolkata, Government of India. No animals were euthanized or sacrificed during any part of the study, and behavioral observations were conducted without any chemical treatment on the individuals. At the end of the experiments, all fish were returned to stock tanks and continued to be maintained in the laboratory.

### Procuring subject animals and maintenance

We used wild-caught zebrafish (from Howrah district, West Bengal, India), bought from a commercial supplier. The fish were maintained in the laboratory in mixed-sex groups of approximately 60 individuals in well-aerated holding tanks (60 × 30 × 30 cm) filled with filtered water. The lighting in the laboratory was maintained at 14 hL:10 hD to mimic the natural LD cycle in zebrafish. They were fed commercially purchased freeze-dried blood worms once a day alternating with brine shrimp *Artemia*. The holding tanks were provided with standard corner filters for circulation. They were maintained in the laboratory for six months before experiments were conducted to ensure they were all adults and were reproductively mature. Holding room temperature was maintained between 23 and 25 °C.

### Experimental setup

**Figure 1:**
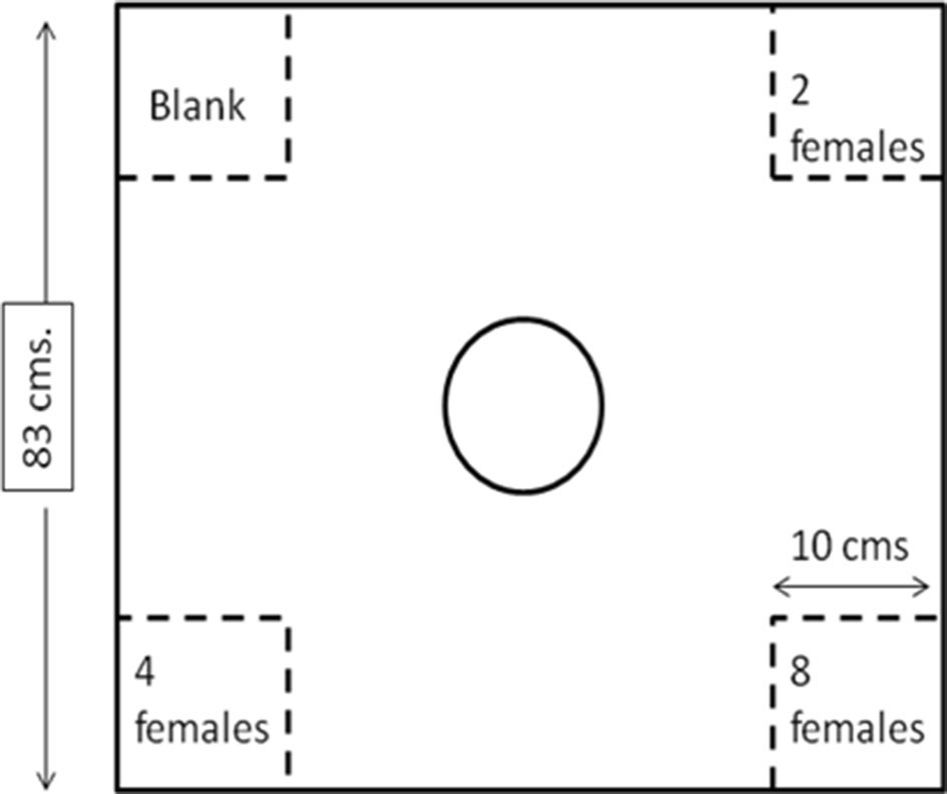
Diagrammatic representation of the arena for the density experimental set-up. The central chamber (indicated by a circle) represents the area where the test males were released and the corner square chamber (separated by transparent mesh) contained females of varying density. The distance of each patch from the central chamber was 40 cm.

The experiments were conducted in a square glass arena (83 × 83 cm), with a half-diagonal of the square from the center that approximated ten fish standard body lengths (i.e. 40 cm, assuming one body length of adult zebrafish to be about 4 cm) (Fig. 1). Each corner of the arena was provided with a square chamber (of sides 10 cm) built from transparent mesh (using synthetic fish nets) for housing the females. This design allowed for the stimuli females to be localized in the patches and not escape into the arena while simultaneously ensuring that the test males can have visuo-chemical communication with the females. The center of the arena was provided with a removable chamber (with holes) for acclimation of the males prior to the trial.

Three sets of experiments were performed to test their association preferences under (1) only varying female densities (2) increasing female and vegetation densities and (3) increasing female densities with decreasing vegetation.

### Association preference experiment with varying female densities

For this experiment, each small chamber within the arena housed two (low number), four (medium number), eight (high number) or no (blank) females. These chambers represented patches of varying female numbers. The position of the female-containing chambers, as well as the composition of females within each patch, was randomized between trials. A total of 20 males were tested for their association preferences. Details on the data collected are provided in Supplementary File S1.

### Association preference experiment with vegetation

**Figure 2:**
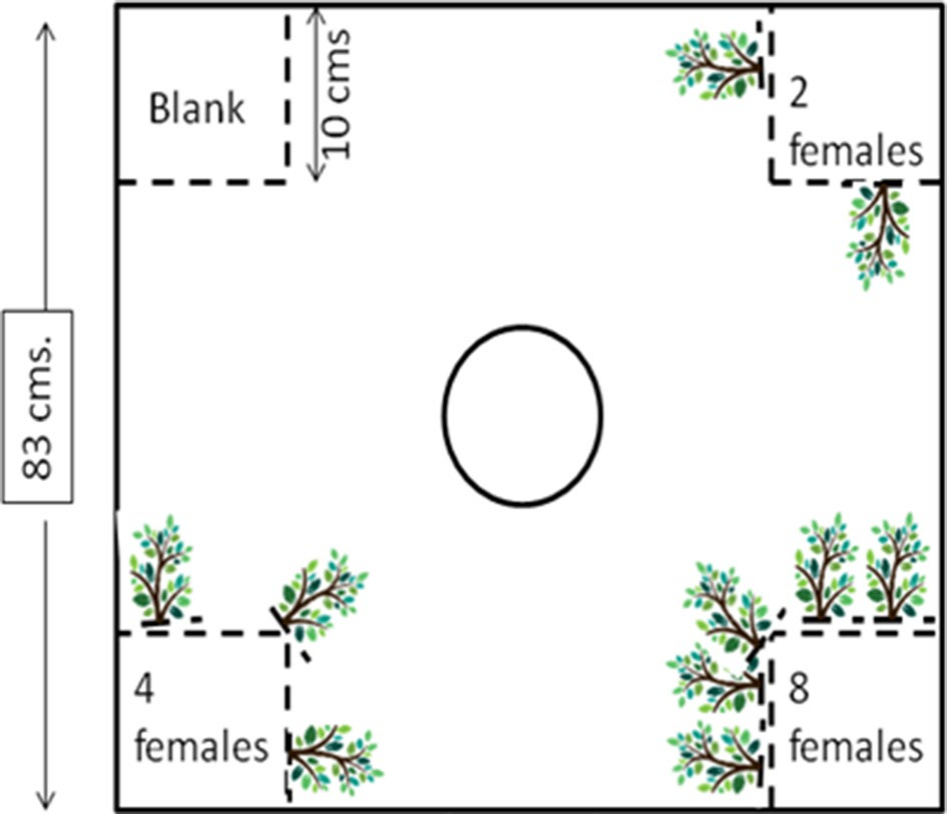
Diagrammatic representation of the arena the vegetation experimental set-up. The central chamber (indicated by a circle) represents the area where the test males were released and the corner square chambers (separated by transparent mesh) contained females of varying density and each patch was associated with variable number of plastic plants representing vegetation cover.

For this experiment, the female-housing chambers (patches) were provided with vegetation (using artificial plants) of varying density (Fig. 2). Each subject fish was tested under two experimental settings. In E1, the number of females was proportional to the density of associated vegetation cover. We used four different densities of females, each associated with different densities of plants

1. one female + no plants (no vegetation—N)
2. two females + two plants (low vegetation—L)
3. four females + three plants (moderate vegetation—M) and
4. eight females + five plants (high vegetation—H).

For E2, we interchanged in the vegetation cover for the two and eight female patches. The patch composition in E2 set were as follows

1. one female + no plants (no vegetation—N)
2. two females + eight plants (high vegetation—H)
3. four females + three plants (moderate vegetation—M) and
4. eight females + two plants (low vegetation—L).

All test males were tested in E1 and E2 on consecutive days in no particular order. Details on the data collected are provided in Supplementary Files S2 and S3.

### Experimental protocol

For the experiment involving association preferences with only varying female numbers a total of 20 males were tested, while 24 males were tested for experiments on the association preferences in varying female numbers combined with vegetation density gradients (E1 and E2 experiments). The experiments were performed two months’ apart to ensure the fish do not retain any memory from the first experiment, and thus they could be treated as two independent sets. We isolated subject males of comparable sizes and kept them in individual isolation in 500 ml jars for four days prior to experiments as that allowed us to keep track of individual fish and also stimulated mate-seeking behavior^21,22^. They were fed freeze-dried blood worms every day at constantly maintained feeding times. The gravid females that were used for the experiment as stimuli for association were isolated (about 22 females) in a small holding tank (30 × 20 × 20 cm) with a feeding regimen similar to the test males. Before the start of each trial, we introduced the females into each chamber (patch) randomly (according to the experimental setup described above) and left them there for 15 min. for acclimation. A single male individual was then gently introduced into the central cylindrical chamber (with a hand-net), open at both ends (made of transparent plastic and provided with holes). After a five-minute acclimation period, the chamber was slowly removed to allow the male to swim freely in the arena and video recording was commenced. Video recordings were done using a camera (Sony DCR-PJ5, Sony DCR-SX22) placed perpendicularly above the arena. The test fish (males and females) were fed only after the end of experimental trials, on each day of experiments. At the end of the trials, the fish were returned to their holding tanks. No subject male fish were tested more than once per experimental setup and trial. The females used for the patches, were housed together (but separate from their male counterparts) in a smaller tank. Before the trials the females were picked randomly and assigned into each patch. During the experiment, the position of females being used was randomized between trials from patch to patch, to avoid the possibility of bias among the subject males for any particular females in the patches.

We recorded the behavior of each test fish for 10 min. All videos were analyzed using the software BORIS^23^. A single visit to any of the patch was denoted when the male approaches within 6 cm (1.5 times their average body length) of the patch. We collected data on three parameters: total number of visits to each patch, the total amount of time spent in each patch and the mean time spent per visit within each patch. The same overall protocol was followed for all sets of experiments.

### Statistical analyses

We noted the total number of visits to each patch, the total duration of time spent in each patch and mean time spent per visit per patch for the entire ten minutes duration of video recording for each test male. We calculated preference index (I) the total number of visits (I_visit) and total time spent (I_time) for each patch as proportion of the total visits made to all four patches^24^.

- I_visit for patch A = No. of visit to patch A/(visit to patch A + visit to patch B + visit to patch C + visit to patch D).
- I_time for patch A = time spent in patch A/(time spent in patch A + time spent in patch B + time spent in patch C + time spent in patch D).

All statistical analyses were performed in R studio (version 1.1.463)^25^. We developed generalized linear mixed models (GLMMs) using package glmmTMB (version 0.2.3)^26^ with ‘fish’ as the random factor and ‘Patches’ as the fixed factor, with four levels representing the four choices for the test (male) fish. Preference for total number of visits (I_visit) as well as total time spent (I_time) were found to fit beta distribution with values ranging between 0 and 1. For data fitting, we added 0.0001 to every value, to remove zeroes. Relevelled models were used to compare the parameters between the four patches. Link = logit was used under beta family to construct the GLMM models.

For analyzing the data for the second and third experiments involving varying female densities along with vegetation densities (E1 and E2), we followed a similar procedure of constructing a GLMM followed by post hoc tests. GLMM models were constructed with a single independent variable, “patch”, that had four levels, designated as H (high vegetation density), M (moderate vegetation density), L (low vegetation density) and N (no vegetation).

## Results

Association preferences of test individuals were measured under two conditions. Firstly, preference of individuals (males) for patches that only varied in terms of female densities were analyzed. Following this, we also analyzed the preferences of males for patches that varied in terms of female densities along with vegetation densities.

### Association preference experiment with varying female densities

**Figure 3:**
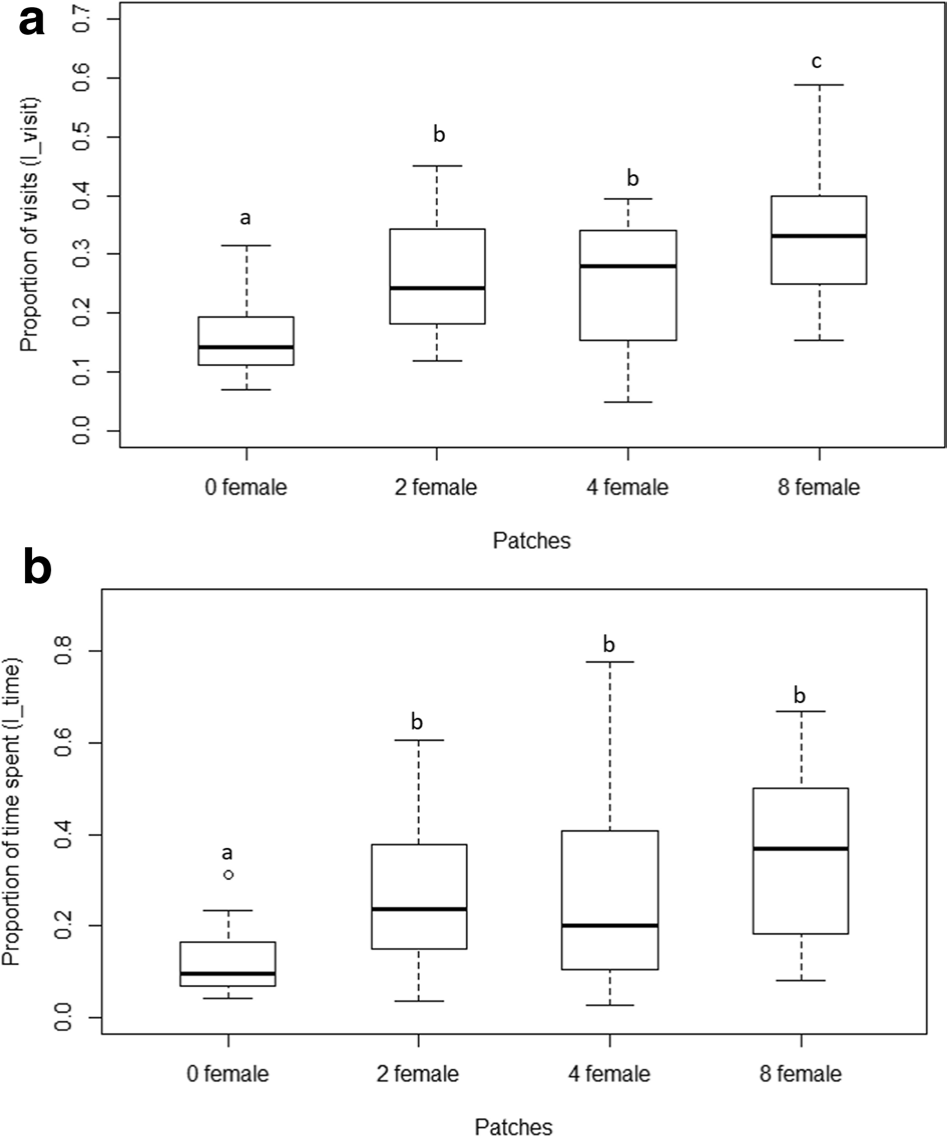
(**a**) Boxplots showing the proportion of visits (I_visit) by the male to the various chambers (patches) containing females in varying density. The chamber containing no females received a significantly lesser number of visits compared to the other three chambers. The eight female patch received higher proportion of visits compared to two and four female patches. Differing alphabets indicate statistical significance (p < 0.05), while similar alphabets indicate no statistical difference. The lower and upper ends of the box plots represent the 1st and 3rd quartiles, the horizontal line within each boxplot is the median, and the upper and lower whiskers are the ×1.5 the interquartile range. Outlines are shown as open circles. (**b**) Boxplots showing proportion of total time (I_time) spent by the male in each of the chambers. Males spent significantly longer time in the three female-containing chambers compared to the chamber with no females. They spent similar proportions of time in 2, 4 and 8 female patches. Differing alphabets indicate statistical significance between the two plots (p < 0.05). The lower and upper ends of the box plots represent the 1st and 3rd quartiles, the horizontal line within each boxplot is the median, and the upper and lower whiskers are the ×1.5 the interquartile range. Outlines are shown as open circles.

The selected prediction model revealed that the fixed factor, ‘patch’ significantly affects the number of visits (Wald type II χ^2^ = 30.33, df = 3, p < 0.01) compared to the null model with only the random factor present (Table 1a). Relevelled GLMM for I_visit showed significant differences between the null patch with the three female-containing patches. Significant difference was found for the eight-female patch compared to four-females (z = 3, p = 0.003) as well as two-female patches (z = 2.07, p = 0.04). There was no significant difference between two and four female patches (z = − 0.94, p = 0.35) (Fig. 3a).

**Table 1:** Summary of results of generalized linear mixed models indicating effect of patch (female number) (fixed factor) and fish id (random factor) on (a) proportion of visits (I_visit) (b) proportion of time spent (I_time).

(a) (8F, 4F, 2F, 0F)				
*Null model*: I_visit ~ (1|FishID) AIC: − 118.9				
*Selected model*: I_visit ~ Patch + (1|FishID) AIC: − 139.7				
Variable	Estimate	Std. error	z-score	p
Intercept	− 1.70	0.19	− 8.76	≪ 0.01
Patch 8F	1.07	0.25	4.31	≪ 0.01
Patch 4F	0.61	0.25	2.39	0.01
Patch 2F	0.68	0.25	2.69	< 0.01

(b) (8F, 4F, 2F, 0F)				
*Null model*: I_time ~ (1|FishID) AIC: − 72.64				
*Selected model*: I_time ~ Patch + (1|FishID) AIC: − 84.58				
Variable	Estimate	z-score	df	p
Intercept	− 1.61	0.13	− 12.21	≪ 0.01
Patch 8F	0.92	0.16	5.42	≪ 0.01
Patch 4F	0.44	0.17	2.52	0.01
Patch 2F	0.59	0.17	3.43	≪ 0.01

The AIC values of selected models along with estimate values, t-scores, degrees of freedom (df) and p values for each factor are shown. p values ≤ 0.05 is considered significant. Patch0F, Patch2F, Patch4F, and Patch8F signify patches with 0, 2, 4, and 8 females, respectively.

Similarly, GLMM constructed for the second parameter, I_time, also showed a significant effect of the fixed factor “Patches” (Wald type II χ^2^ = 18.71, df = 3, p < 0.01) (Table 1b) when compared to the corresponding null model. We found that I_time was significantly lower in the null patch (i.e. patch with no females) compared to the other three patches containing females. However, there was no significant difference between the two, four and eight female containing patches for the proportion of time spent in each patch (Fig. 3b).

### Association preference experiment with varying female and vegetation densities

We first tested the association preferences of test individuals to patches with increasing female and vegetation densities (E1) and then tested their preferences for patches under contrasting female and vegetation densities (E2).

#### For E1 set

**Figure 4:**
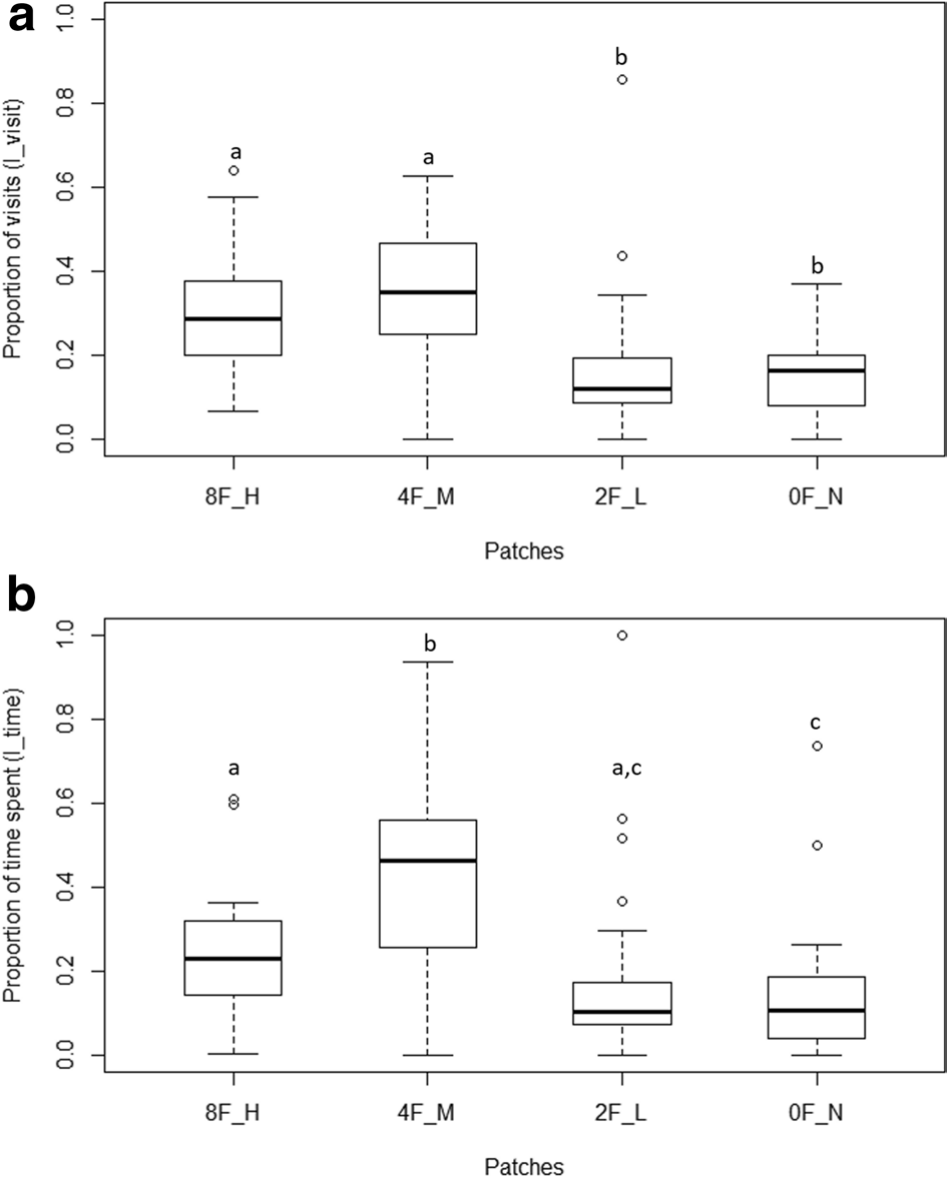
(**a**) Boxplots showing the proportion of visits (I_visit) by the male to the various chambers containing the females in varying density in vegetation presence (E1 set). High (H) and medium (M) chambers having eight females (and high plant density) and four females (and medium plant density) respectively had a higher proportion of visits by the male compared to the low (L) (two females and low plant density) and null (N) (one female and no plants) chambers. Similar alphabets placed above the plots indicate no statistical difference whereas dissimilar alphabets indicate significant statistical difference (p < 0.05). The lower and upper ends of the box plots represent the 1st and 3rd quartiles, the horizontal line within each boxplot is the median, and the upper and lower whiskers are the ×1.5 the interquartile range. Outlines are shown as open circles. (**b**) Boxplots showing the proportion of time (I_time) spent by the male in each of the chamber (E1 set). Males spent a significantly greater proportion of time in medium (M) chamber compared to the other three patches. Similar alphabets placed above the plots indicate no statistical difference whereas dissimilar alphabets indicate significant statistical difference (p < 0.05). The lower and upper ends of the box plots represent the 1st and 3rd quartiles, the horizontal line within each boxplot is the median, and the upper and lower whiskers are the ×1.5 the interquartile range. Outlines are shown as open circles.

The selected prediction model revealed that the fixed factor ‘patch’ significantly affects the number of visits (Wald type II χ^2^ = 73.39, df = 3, p < 0.01) compared to the null model with only the random factor present (Table 2a). Relevelled GLMM for I_visit showed significant differences between H patch with L (z = 3.12, p = 0.001) and N (z = 3.4, p = 0.0007) patches. Proportion of visits to M patch were also significantly greater than L (z = 4.35, p < 0.001) or N (z = 4.63, p < 0.001) patches. I_visit was comparable for H and M patches (z = − 1.2, p = 0.22) (Fig. 4a).

**Table 2:** Summary of results of generalized linear mixed models indicating effect of patch (female number + vegetation density) and fish id (random factor) on (a) proportion of visits (I_visit), (b) proportion of time spent (I_time), in E1 set.

E1 set: (a) (H + 8F, M + 4F, L + 2F, N + 1F)				
*Null model*: I_visit ~ (1|FishID) AIC: − 64.69				
*Selected model*: I_visit ~ Patch + (1|FishID) AIC: − 86.05				
Variable	Estimate	z-score	df	p
Intercept	0.35	0.03	12.04	≪ 0.01
Patch H	− 0.05	0.04	− 1.22	0.22
Patch L	− 0.18	0.04	− 4.35	≪ 0.01
Patch N	− 0.19	0.04	− 4.63	≪ 0.01

E1 set: (b) (H + 8F, M + 4F, L + 2F, N + 1F)				
*Null model*: I_time ~ (1|FishID) AIC: − 5.42				
*Selected model*: I_time ~ Patch + (1|FishID) AIC: − 24.25				
Variable	Estimate	z-score	df	p
Intercept	0.42	0.04	10.48	≪ 0.01
Patch H	− 0.18	0.05	− 3.18	≪ 0.01
Patch L	− 0.24	0.05	− 4.29	0.01
Patch N	− 0.28	0.05	− 4.86	≪ 0.01

Proportion of time spent with each group was significantly affected by the fixed factor ‘patch’. The selected prediction model revealed that the fixed factor ‘patch’ significantly affects the number of visits (Wald type II χ^2^ = 70.2, df = 3, p < 0.01) (Table 2b). I-time was significantly higher in M patch compared to the other three (M–H: z = 3.1, p = 0.001; M–L: z = 4.3, p < 0.001; M–N: z = 4.6, p < 0.001) (Fig. 4b).

#### For E2 set

**Figure 5:**
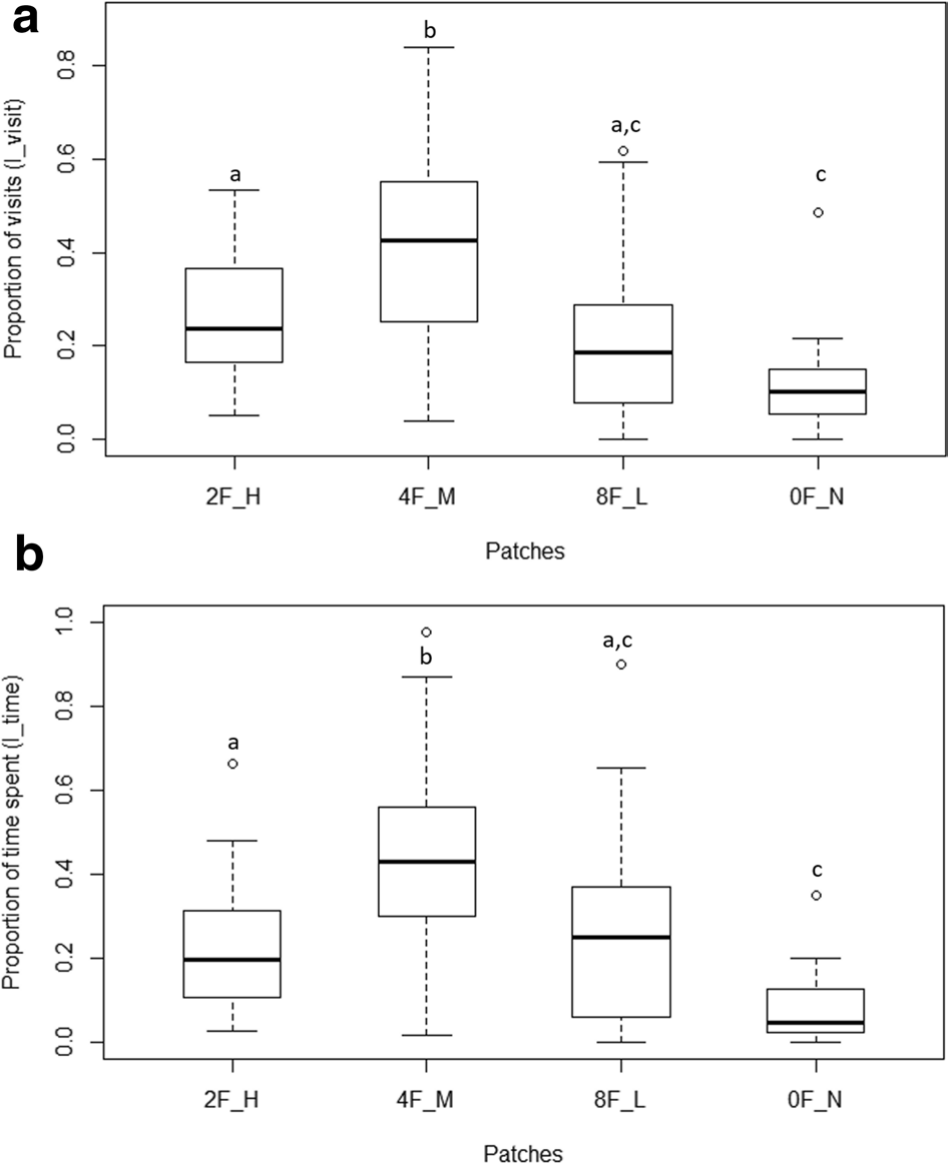
(**a**) Boxplots showing the proportion of visits by the male to the various chambers (E2 set). Males visited the medium (M) chamber (four females and medium plant density) significantly more than the high (H) (two females with high plant density), low (L) (eight females with low plant density) or Null (N) (one female with no plants) chambers. H patch also had greater proportion of visits compared to N patch. Similar alphabets placed above the plots indicate no statistical difference whereas dissimilar alphabets indicate significant statistical difference (p < 0.05). The lower and upper ends of the box plots represent the 1st and 3rd quartiles, the horizontal line within each boxplot is the median, and the upper and lower whiskers are the ×1.5 the interquartile range. Outlines are shown as open circles. (**b**) Boxplots showing the proportion of time spent (I_time) by the male in each of the chamber (E2 set). The test males spent statistically greater proportion of time in M patch compared to the other three patches. H patch also had greater proportion of time spent compared to N patch. Similar alphabets placed above the plots indicate no statistical difference whereas dissimilar alphabets indicate significant statistical difference (p < 0.05). The lower and upper ends of the box plots represent the 1st and 3rd quartiles, the horizontal line within each boxplot is the median, and the upper and lower whiskers are the ×1.5 the interquartile range. Outlines are shown as open circles.

The selected prediction model revealed that the fixed factor ‘patch’ significantly affects the number of visits (Wald type II χ^2^ = 46.11, df = 3, p <  < 0.01) compared to the null model with only the random factor present (Table 3a). I_visit was significantly higher for M patch compared to H (z = 3.2, p = 0.001), L (z = 4.7, p < 0.001) and N (z = 6.5, p < 0.001) patches. Proportion of visits to H patch was also significantly greater than N (z = 3.2, p = 0.001) patch. (Fig. 5a).

**Table 3:** Summary of results of generalized linear mixed models indicating effect of patch (female number + vegetation density) and fish id (random factor) on (a) proportion of visits (I_visit), (b) proportion of time spent (I_time), in E2 set.

E2 set: (a) (H + 2F, M + 4F, L + 8F, N + 1F)				
*Null model*: I_visit ~ (1|FishID) AIC: − 36.15				
*Selected model*: I_visit ~ Patch + (1|FishID) AIC: − 67.81				
Variable	estimate	z-score	df	p
Intercept	0.41	0.03	12.82	≪ 0.01
Patch H	− 0.15	0.04	− 3.27	< 0.01
Patch L	− 0.21	0.04	− 4.73	≪ 0.01
Patch N	− 0.30	0.04	− 6.55	≪ 0.01

E2 set: (b) (H + 2F, M + 4F, L + 8F, N + 1F)				
*Null model*: I_time ~ (1|FishID) AIC: − 5.3				
*Selected model*: I_time ~ Patch + (1|FishID) AIC: − 38.21				
Variable	estimate	z-score	df	p
Intercept	0.44	0.04	11.78	≪ 0.01
Patch H	− 0.22	0.05	− 4.09	≪ 0.01
Patch L	− 0.20	0.05	− 3.73	≪ 0.01
Patch N	− 0.37	0.05	− 6.88	≪ 0.01

Proportion of time spent with each group was significantly affected by the fixed factor ‘patch’. The selected prediction model revealed that the fixed factor ‘patch’ significantly affects the number of visits (Wald type II χ^2^ = 47.99, df = 3, p < 0.01) (Table 3b) I-time was significantly higher in M patch compared to the other three (M–H: z = 3.1, p < 0.001; M–L: z = 4.2, p < 0.001; M–N: z = 4.9, p < 0.001). Proportion of time spent in H patch was also significantly greater than N (z = 2.79, p = 0.005) patch (Fig. 5b).

## Discussion

Our study aimed to understand the influence of female shoal size on association preference in wild male zebrafish. Using a multi-choice experimental design, we also tried to explore the interplay between shoal size and vegetation cover. In general, males showed a preference for female-containing patches and this preference was found to be greater for patches with more females. Results from our experiments with inclusion of additional factors such as vegetation, however, indicated that importance of presence of ecological factors such as vegetation can take precedence over female densities. We discuss our findings in detail below.

In our first experiment with varying female numbers, we found that males clearly prefer a patch with females over the null or zero-female containing patch. Indeed, it has been reported earlier in two-choice tests as well that zebrafish prefer a compartment with a single fish than one without any^11^. In our experiment, we found that among the three remaining patches, the males’ visit to the 8-female patch was significantly higher in proportion, in comparison to either the 2 or 4-female patches. It lacked any specific preference towards the later two patches themselves. Furthermore, the second preference parameter (I_time) revealed that all three female-containing patches had similar proportions of time spent by males near them. Male zebrafish have been reported to lack inherent preferences for a larger shoal unlike the females^9–11^. Our results seem to show that while the males prefer to visit the 8-female patch more often, they end up spending similar times across all the three group sizes. The high density of females in that patch, might act as a strong attractive stimulus for the males but not enough to sustain every visitation for a longer period of time. Indeed, a previous study on zebrafish does show that males preferred to shoal with female compared to male shoals, but showed no preference for larger shoals over smaller shoals, irrespective of whether they were composed of males or females^7^.

Some interesting patterns emerged when we incorporated artificial vegetation as an additional factor to group size variation. Firstly, in both E1 and E2 sets, high and medium density vegetation patches were preferred by the males. In E2, when only 2 females were present in the high vegetation patch, we still see that it is preferred over a low vegetation patch with eight females. In terms of the proportion of visits (I_visit), we found no particular preference between high and medium vegetation patches in E1. But in E2, where M patch had four females and H patch had 2, we see M being visited more often than H. This is in contradiction to the finding of the first experiment where we saw the males had no preference between 4 and 2-female groups. Males are able to discriminate between the two group sizes and show a preferential association only in presence of artificial vegetation. In terms of I_time, males again show significant preference for M patch over H in E2 condition. Interestingly, they continue to spend greater proportion of time in M patch over H even in E1 set, where the latter had eight females. One possibility could be that high vegetation density might not allow for the males to assess the H patch properly, especially when it housed eight females, due to the high density. Thus, we can see that vegetation influences shoal association in male zebrafish. We also see a complex interaction between female number and vegetation density, possibly influencing the cost–benefit assessment for associating with a group.

In the wild, shoaling decisions are shaped by the inherent preferences of the individuals but as well as ecological factors. Multiple ecological factors work concomitantly influencing the cost–benefit trade-off for shoaling decisions and shoaling behavior. Temperature is another known regulator of shoaling decisions in zebrafish^10^. We still have limited understanding of how vegetation itself regulates shoaling behavior in different species. Bhat et al.^20^ found that shoaling distance between members in a zebrafish group depended on vegetation presence in wild-caught as well as lab-bred populations. Floating vegetation also regulates the amount of sunlight penetrating into the water. UV light is a known regulator of shoaling decisions in fish like three spined sticklebacks^27^. Littoral decomposition of aquatic vegetation also imparts chemical cues that influence shoal cohesion^28^. However, to the best of our knowledge this is the first elaborate examination of the role of aquatic flora in determining shoaling preferences in zebrafish, using an experimental paradigm that using a gradient in female as well as vegetation densities. Our results revealed that preference for female-group sizes can vary depending on associated ecological factors. It would be further interesting to explore how the preferences we observed, change for all male and mixed-sex ratio groups. We also require further experimentation to tease apart the role of vegetation and group size on association preferences.

Most of the prior work on zebrafish shoaling preferences had been conducted on lab-bred strains or fish obtained from the pet store. In contrast, our work involved measuring shoaling preference in wild-caught zebrafish, which would be a better representative of the natural behavioral phenotypes of wild zebrafish. Furthermore, we used a novel a multi-choice experimental arena instead of a traditional two-choice design. Our design allowed for presenting multiple stimuli simultaneously to explore their preferences in-depth. In future studies, incorporation of other ecological factors in a multi-choice setting can shed further light in understanding how shoaling behavior is regulated across sexes.

## Supplementary Information

Supplementary Information S1.

Supplementary Information S2.

Supplementary Information S3.

## Data Availability

All data generated or analysed during this study are included in this published article (as Supplementary Information Files S1, S2, S3).
